# Porous high-density polyethylene in facial reconstruction and revision rhinoplasty: a prospective cohort study

**DOI:** 10.1186/1746-160X-8-17

**Published:** 2012-05-29

**Authors:** Shabahang Mohammadi, Shadi Ghourchian, Farzad Izadi, Ahmad Daneshi, Aslan Ahmadi

**Affiliations:** 1 Ear Nose Throat (ENT) and Head and Neck Surgery Research Center, Hazrat Rasoul Akram Hospital, Tehran University of Medical Sciences, Sattarkhan st, 1445613131, Tehran, Iran; 2 Students’ Scientific Research Center of Tehran university of medical sciences (SSRC), Tehran University of medical sciences, Tehran, Iran

**Keywords:** Medpor, Rhinoplasty, Frontal reconstruction, Reconstruction of orbital rim

## Abstract

**Introduction:**

Initial methods which used human tissues as reconstruction materials caused different problems including rejection, limited shapes and infection. In 1970s, PHDPE (Medpor®) was introduced by its exclusive advantageous including no donor site morbidity, easily shaped and the minimal foreign body reaction. Hereby, we report our experience of using Medpor® in facial reconstruction especially in frontal reconstruction and orbital rim with a large sample size.

**Methods:**

This study was a prospective cohort study. Surgical techniques included using Medpor® in reconstruction of lamina papiracea (LP) (15 patients), frontal bone (15 patients), orbital rim (18 patients) and open rhinoplasty (8 patients). All interventions on LP were performed by endoscopic procedures. All frontal operations were carried out by bicoronal incision. In orbital defects, we used subciliary incision.

**Results:**

From all 56 patients, 1 case had primitive neuroectodermal tumor (PNET) of maxillary sinus. In that case, reconstruction of inferior orbital rim was not successful and extrusion was occurred after radiotherapy. In rhinoplasty and other experiences no extrusion or infection were detected within the next 1 to 3 years of follow up. There were not any palpable and visible irregularities under the skin in our experiences.

**Conclusions:**

In this study the patients did not experience any complications during the follow up periods and the satisfaction was remarkable. Gathering these data gives rise to future review studies which can provide more organized evidences for replacing classic reconstructive methods by the presented material.

## Introduction

Loss of tissue caused by trauma, cancer or surgery usually needs some materials for reconstruction. Initial methods which used human tissues as reconstructive materials caused different problems including disable to shape the graft, limitation of accessibility and donor site morbidity [1-3].

In 1828, using artificial materials was established by using gold in nose reconstruction [1,4].

Using silicon rubber, polyamide and Gore-Tex has been improved since 1950, but each of them was accompanied by different reactions such as extrusion, infection and not well-shaped slippery reconstruction [4-9].

In 1970s porous high-density polyethylene (PHDPE, Medpor®) was introduced by its exclusive advantageous including no donor site morbidity, easily shaped and the minimal foreign body reaction [10-13]. Furthermore the likelihood of infection was reduced by significant vascularization of the tissue within one month [14,15] (was seen in animals [12,16]) and the proliferation of the surrounding tissue (was seen in humans) [17]. Auricular reconstruction, augmentation of malar, chin, nasal dorsal areas, and restoration of the inferior orbital rim or orbital floor were previously reconstructed by Medpor [18-20].

In revision rhinoplasty, sufficient cartilage in septum is not usually exists to be used for reconstructing. Lack of cartilage tissue gives rise to harvest an extra cartilage from rib or auricle. This procedure elongates the time of surgery and also has many cosmetic problems, so the use of an artificial material with limited complications is considered as a choice in this situations.

Hereby, we report our experience of using Medpor® in some aspects of facial reconstruction especially in frontal reconstruction and rhinoplasty which were not paid enough attention in other studies, . Also to understand the probable complications of this recent material, the patients were followed for a long period. Previous studies were performed with small sample sizes and this study was designed to make some new organized evidences for adding to previous experiences.

## Methods and materials

### Ethical approval

This study was a prospective cohort study approved by the institutional review board of the ENT research center of Rasul-e-Akram Hospital, Tehran University of Medical Sciences (TUMS) and started on 2008. Before starting the study all the authors were informed about the procedure and the aim of the study. All of the patients were informed by the authors about the method and the usage of material, its benefits and probable complications. Also all accessible procedures which could be used for reconstruction and their own disadvantageous were explained to the patients. Prior to the operation, a written consent form was signed by the patients or their own families.

### Patients

Of patients referred to the ENT ward due to the lamina papiracea (LP) impairment, who had enophtalmy or bony fracture was entered to the study and patients with visual acuity defect or eye movement problems were excluded by an ophthalmologist consult. Also patients with frontal trauma were included if there was a bony defect and were excluded if a soft tissue defect especially associated with a near infected ulcer was detected. Patients with depressed frontal fracture underwent classic open surgery because of future irreparable cosmetic problems following using new methods in these complicated patients. To use Medpor ® in rhinoplasty procedures, patients with deviated nose and who underwent a previous rhinoplasty were excluded due to the lack of septal cartilage which was needed for implanting the material.

The clinical characteristics included gender, age, initial diagnosis, co-morbidity and complications which were entered to a self-designed check list before and after the surgery.

### Surgical techniques

#### Lamina papiracea

In our study, in the experience of reconstructing LP, 15 patients with traumatic fracture of LP with any kinds of cosmetic and functional problems like enophthalmy and hypophthalmy underwent the endoscopic procedures. In this method, after shrinkage of nasal cavity, the injection of epinephrin with lidocaine at the axilla of middle turbinate and uncinate process 1/100000 was performed to minimize bleeding during the operation. Then anterior ethmoidectomy was performed to expose the fractured LP and the defect . After that, we cut and shaped Medpor® titanium plate (10 × 10 mm) as the same size of the defect. After reduction of herniated orbital or periorbital fat, we rolled the plate and transferred it through the defect of LP over the periorbita and under the periost of LP. Then we packed the vestibule for 3 days after the surgery.

#### Frontal reconstruction

In 15 patients with traumatic frontal fracture we used Medpor® to reconstruct the anterior table of frontal bone.

In this method, we used bicoronal incisions in the location of 2 cm superior of the hairline and dissected it in subplatismal plane above the fracture line and then the plane of the dissection was changed to the subperiost.

The particle of the fractured bone was taken out of frontal sinus and then Medpor® titanium plate was curved like other sides of forehead and fixed by the surrounding tissues.

#### Orbital reconstruction

In reconstruction of inferior orbital rim in 18 cases we used subciliary incision. Elevation of orbicularis occuli was done in preseptal plane and at the inferior border of orbit changed the plane to the subperiost at the level of fracture. In the patients with large gap on the inferior orbital wall with post traumatic hypophthalmia, the plate of Medpor® titanium was fixed at subperiosteal plane of inferior orbital wall with single suture to the periost of anterior face of maxilla.

#### Open procedure in revision rhinoplasty

In our study we used open procedure for using Medporc titanium in revision rhinoplasty surgery in 8 patients. In these patients we used Medpor® as collumelar strut to have good projection and rotation in the patients with tip ptosis. These plates were fixed with 6–0 nylon to the medial crura of both lower lateral cartilages. We also used Medpor® as only lower lateral cartilage graft in 5 patients. We put these plates in pocket over the cartilages through marginal incision and sutured the incision. This technique used to augment the cartilage in patients with pinching of lower lateral cartilage.

#### Follow up

In this study, patients’ improvements were passively checked by the surgeons. The more important criteria to describe satisfaction of the surgery were based on the clinical follow up by the surgeons that revealed no extrusion, asymmetry, bone depression and irregularity.

Patients with frontal reconstruction were followed by the surgeons for 3 years (within different intervals based on the initial complications). They were checked for asymmetry, irregularity, bone depression and extrusion by inspection and touching. Patients who underwent rhinoplasty were checked for extrusion for 2–2.5 years by endoscopic procedures. Others with orbital reconstruction were followed for 1 year and evaluated for enophtalmy or hypophtalmy improvement and asymmetry which were considered as the satisfaction criteria in this group.

## Results

From 56 patients, 15 underwent reconstruction of LP, 15 underwent frontal reconstruction, 18 underwent orbital reconstruction, and 8 underwent open surgery for revision rhinoplasty. The mean age of the patients was 36.2 ± 7.34. Of 56 patients, 42(75%) were men.

All interventions on LP were performed by endoscopic procedures regardless of the basic problem (cosmetic or functional). From 15 patients who underwent frontal reconstruction, all of them were referred because of trauma. All frontal operations were carried out by bicoronal incision. In all of the 18 patients who were suffered from orbital defect, we used subciliary incision. From all 56 patients, 1 case had primitive neuroectodermal tumor (PNET) of maxillary sinus in that reconstruction of inferior orbital rim was failed and extrusion was occurred after radiotherapy. He was a 26-year-old man who was presented with extrusion after 6 months. To repair the inferior orbital rim in that patient, a reconstructive surgery was performed after 1 year of the failure by using paramedian forehead flap.

In rhinoplasty and other experiences we did not face to any extrusions or infections. There were not any palpable and visible irregularities under the skin after the operation.

## Discussion

In this study we reported our experience of using Medpor® in 56 patients who suffered from facial impairments. The defects were categorized into 4 groups with different surgery strategies with the lowest visible scar formations. The patients were followed for 1 to 3 years which did not reveal any complications and the satisfaction was remarkable. Extrusion occurred just in 1 patient with maxillary PNET following radiotherapy.

Since 19th century, autologous tissues were used for cosmetic purposes. Tibia bone was used as a reconstructive tissue for nose by Israel [1-3]. Those methods were accompanied by lots of time for surgery and the problems with shaping the graft such as the impaired organ like nose, the limitation for availability and high morbidity rates of donors. Besides that, those homologous tissues were resorbed in different experiences [1,4,21-23]. Also there was a fear of blood transferring diseases by using homologous tissues [8]. After those decades, allogenic grafts were rejected because of the infection [24].

The procedure of using artificial materials was initialed by Roussett, in 1828. He used gold for nose reconstruction [1,4,8,25,26]. Following that Joseph used ivory for this purpose in 1900.

The expressed materials were not used anymore due to poor tissue tolerance [1,8]. Since 1950 silicon rubber, polyamide, Gore-Tex started to be used with their specific reactions. In 1953 Brown used silicon. This substance usually was surrounded by fibrous capsule and predisposed to be extruded by host tissue within a long time, and also the absence of proper vascularization [4-9].

Extrusion risk was decreased by using polyamide mesh. In this process, fixing the material was caused by in growth of fibrous tissue within several months. Despite these advantageous, severe inflammatory responses were raised by the recipient body [1,5,6]. Although Gore-Tex or polytetrafluoroethylene (ePTFE) was not accompanied with extrusion or degradation, its elasticity was not enough to be shaped properly for the nose. Also it remained slippery due to the lack of host tissue in growth [5,6,27].

One of the other artificial materials for reconstruction, Proplast, was not suggested due to the antigenic reaction and the increased risk of infection [16,28].

By developing in using synthetic materials, PHDPE (Medpor®) was introduced to be used for cosmetic defects in 1970 [11-13].

Synthetic materials can be shaped and provided sterile in the factory. Also there is no donor site morbidity [10]. PHDPE made of high density and pure polyethylene, can be shaped easily in the room temperature, causes minimal foreign body reaction and so can be tolerated easily by the host tissue. Capsule formation [12,13,29,30] is noticeable in reconstructing with PHDPE and also it is implanted constantly by the host tissue in growth [5,31].

Rapid in growth of vascularized tissue with collagen deposition stabilizes Medpor® and decreases the probability of infection and movement due to the contraction of surrounding host tissues [12].

The efficacy and advantages of Medpor® had been investigated among patients with primary orbital trauma [1,30,32,33].

In 1990 C. S. Maas et.al compared the gross and microscopic response to implanted materials used for facial bone augmentation in dogs. They revealed that the failure or success of the graft is associated with the mass of the surrounding tissues that support implant movement. Among them, solid and porous alloplastic showed proper tissues response but none of them could be stable on the underlying bone [34].

Jane M, et al. in 2003 revealed that vascularization appeared even in the relatively avascular subperiosteal space after reconstruction with porous polyethylene. By their own theory vacuolization could be justified by the link between sinus mucosa and the implant which could appear in the fracture. They saw giant cell inflammation in all of their cases [34].

In our study, we used Medpor® for the defects of LP by endoscope procedures, and also the defects of frontal bone, orbital rim and rhinoplasty by open surgery. Except one case with PNET of maxillary sinus, other patients were not involved by any problems of using Medpor®.

Due to the proper vascularization and also good prophylactic antibiotic administration, there were no infectious and rejection in our patients. All of the patients were consent within 3 years after the procedure. The patients, who underwent open surgery, didn’t face to any complications including visible irregularity and the scar of the surgery.

Using new instruments in facial reconstruction should be associated with enough attention to future cosmetic aspects especially asymmetry and irregular feature of the reconstructed tissue because reoperation of prosthesis cannot be usually provided following the fibrosis and tissue adherence. This precision makes new methods to be avoided by some rookie surgeons. In our study, the high prevalence of eye movement disorders among patients with orbital rim fracture, limited the number of our cases. Also some patients disagreed to be entered the study because they could not follow the follow up schedules. Also some patients needed emergent surgeries while the expert man was not the on time resident surgeon to evaluate the patient for entering to the study.

## Conclusions

Considering the result of our study about reconstructing the orbital rim and frontal bone by using Medpor®, and also the acceptable result about rhinoplasty like other similar studies, it seems that initial surgical methods can be replaced by different procedures with Medpor®. Gathering these data gives rise to future review studies which can provide more organized evidences for replacing classic reconstructive methods by the presented material. The advantageous of Medpor® should be explained to all patients. Also more studies with the purpose of comparing 2 groups (1 group can use initial methods, and the other use Medpor®) are suggested.

## Abbreviations

PHDPE, Porous high-density polyethylene; LP, Lamina papiracea; PNET, Primitive neuroectodermal tumor; TUMS, Tehran University of Medical Sciences.

## Competing interests

We declare that we had no competing interests.

Financial competing interests

· In the past five years have you received reimbursements, fees, funding, or salary from an organization that may in any way gain or lose financially from the publication of this manuscript, either now or in the future? Is such an organization financing this manuscript (including the article-processing charge)? No.

· Do you hold any stocks or shares in an organization that may in any way gain or lose financially from the publication of this manuscript, either now or in the future? No.

· Do you hold or are you currently applying for any patents relating to the content of the manuscript? Have you received reimbursements, fees, funding, or salary from an organization that holds or has applied for patents relating to the content of the manuscript? No

· Do you have any other financial competing interests? No

Non-financial competing interests

Are there any non-financial competing interests (political, personal, religious, ideological, academic, intellectual, commercial or any other) to declare in relation to this manuscript? No.

## Authors’ contributions

SM: has made substantial contributions to conception and design, or acquisition of data, designing the study, surgery (rhinoplasty and orbital rim and frontal reconstruction 2) has been involved in revising the manuscript and 3) has given final approval of the version to be published. AA (Corresponding author): has made substantial contributions to conception and design, or acquisition of data, designing the study, surgery (rhinoplasty and orbital rim and frontal reconstruction 2) has been involved in revising the manuscript and 3) has given final approval of the version to be published. FI: has made substantial contributions to conception and design, or acquisition of data, designing the study, surgery (rhinoplasty and orbital rim and frontal reconstruction 2) has been involved in revising the manuscript and 3) has given final approval of the version to be published. SG: 1) acquisition of data, and analysis of data; 2) has been involved in drafting the manuscript and revising it critically for important intellectual content and 3) have given final approval of the version to be published. All authors read and approved the final manuscript.

## Conflict of interest

Hereby we confirm that this article is not reviewing by other journals. There is no conflict of interest.

All financial supports were provided by Ear-Nose-Throat [ENT] and Head and Neck research center of Rasul-e- Akram Hospital, Tehran university of medical sciences, Tehran, Iran.
